# MicroRNA expression profiles in sinonasal biopsies to support diagnosis of granulomatosis with polyangiitis

**DOI:** 10.3389/fimmu.2025.1579750

**Published:** 2025-04-14

**Authors:** Milanka Živanović, Alojzija Hočevar, Nina Zidar, Metka Volavšek, Luka Bolha

**Affiliations:** ^1^ Institute of Pathology, Faculty of Medicine, University of Ljubljana, Ljubljana, Slovenia; ^2^ Department of Rheumatology, University Medical Centre Ljubljana, Ljubljana, Slovenia; ^3^ Faculty of Medicine, University of Ljubljana, Ljubljana, Slovenia

**Keywords:** granulomatosis with polyangiitis, sinonasal tissue, microRNA, inflammation, biomarker

## Abstract

**Objectives:**

To identify aberrantly expressed microRNAs (miRNAs) in sinonasal tissue biopsies of patients with granulomatosis with polyangiitis (GPA), associate their expression profiles to sinonasal histopathology, and assess their differential expression between subgroups of clinically proven GPA patients, healthy controls and patients exhibiting inflammation of other etiology.

**Methods:**

We included formalin-fixed, paraffin-embedded biopsy tissue samples of sinonasal mucosa from 37 patients with clinically proven GPA, 15 patients with inflammation of other etiology and 14 control patients with normal histology. Of the included GPA patients, 20 patients had characteristic GPA-related histological features, while 17 patients displayed non-specific GPA histopathology in their sinonasal biopsy. Assessment of histological parameters was performed using histopathological techniques, and analysis of miRNA expression with miRCURY LNA miRNA miRNome Human PCR Panels and quantitative real-time PCR.

**Results:**

We determined expression of 306 miRNAs in sinonasal biopsy samples, which displayed different extent of dysregulation between individual patient groups. Based on their potential to discriminate between the controls, non-GPA and GPA patient subgroups, dysregulation of 11 miRNAs was further assessed, of which miR-1-3p/-21-3p/-93-5p/-155-5p/-1248/-31-3p/-182-5p/-183-5p and let-7b-5p held the potential to stratify patients based on their sinonasal tissue miRNA profile. Notably, several of these miRNAs were associated with the presence of granulomas, vasculitis and necrosis in sinonasal biopsies of GPA patients.

**Conclusion:**

Our study identifies novel miRNAs putatively implicated in the pathogenesis of GPA, and highlights dysregulated miRNAs as supporting biomarkers in establishing GPA diagnosis, particularly in the early phases of the disease, or in patients with atypical GPA presentation.

## 1 Introduction

Vasculitides associated with antibodies against the components of the cytoplasm of neutrophil granulocytes (i.e., anti-neutrophil cytoplasmic antibodies; ANCAs) are rare small-vessel vasculitides with a complex and not yet fully elucidated etiopathogenesis (1–3). Globally, their incidence is increasing, which could be attributed to several factors, including an improved clinical recognition, advances in imaging and molecular diagnostics (4). Within the ANCA-associated vasculitis (AAV) spectrum, three genetically distinct but clinically overlapping diseases are distinguished: granulomatosis with polyangiitis (GPA), microscopic polyangiitis (MPA), and eosinophilic granulomatosis with polyangiitis (EGPA) (1–3, 5, 6). GPA is clinically characterized by a triad of upper and lower respiratory tract, and renal involvement (7–9). Histologically, characteristics of GPA are necrotizing vasculitis of small- and medium-sized vessels, tissue necrosis, granulomatous inflammation of the airways and necrotizing pauci-immune glomerulonephritis (5, 7, 8, 10). GPA is typically associated with anti-proteinase 3 (anti-PR3) antibodies, showing a cytoplasmic immunofluorescence staining pattern (c-ANCA), and ANCAs are detected in more than 95% of patients (3, 6, 9–11).

The head and neck region and the upper respiratory tract are regarded as a common site of the onset of GPA (12, 13), and a sinonasal biopsy may enable diagnosis in the early phases of the disease, or in patients with atypical presentation of GPA. As such, a positive biopsy of the sinonasal mucosa, or other sites in the upper respiratory tract, has a high positive predictive value of up to 100%, indicating few or even no false positive results (14). However, upper respiratory tract biopsies, even in patients with clinically confirmed GPA, often lack diagnostic granulomas and vasculitis, and show only non-specific inflammation. Therefore, there is a necessity for more sensible diagnostic biomarkers other than simple morphological features in the diagnosis of the early phase of GPA, which is crucial for treatment and prognosis of affected patients (9).

Over the past 20 years, a prominent role of epigenetics in the pathogenesis and pathophysiology of systemic autoimmune diseases has been demonstrated, particularly in the field of microRNA (miRNA) research. miRNAs are a class of small (~18–23 nt long) non-coding RNAs (ncRNAs), involved in posttranscriptional regulation of gene expression through translational inhibition or degradation of their target messenger RNAs (mRNAs). Dysregulated miRNA expression can lead to aberrant immune function and has been implicated in the development and sustaining of various inflammatory and autoimmune diseases, including vasculitides (15–19). Depending on the pathological context, changes in miRNA expression patterns can be detected in specific cell types, tissues, organs and extracellular fluids, and may associate with the patient age, developmental stage of an organism, environmental factors and disease states. Therefore, dysregulated miRNAs hold great potential to serve as biomarkers, especially since their dysregulation often reflects well the underlying disease state and/or specific disease features (20).

An overall insight into the role of miRNA dysregulation in the pathogenesis of GPA remains limited. To date, miRNA dysregulation has been assessed by expression profiling experiments in renal tissues and small urinary extracellular vesicles (EVs) of patients with AAV (21, 22), whereas, aberrant expression of several miRNAs has been also determined in circulating neutrophils, circulating EVs and peripheral blood regulatory T cells (Tregs) from patients with GPA (23–25). Studies have also revealed a dysregulated miRNA status in nasal tissue of patients with GPA (26–28). However, identification of GPA-specific miRNA signatures in sinonasal biopsy tissues that would enable discrimination of patients with characteristic GPA histological features from patients characterized by non-specific GPA histopathology and inflammatory conditions of other etiology has not been fully addressed.

In this study, we therefore aimed to identify miRNAs whose expression profiles discriminate between sinonasal mucosa of patients with GPA, patients with inflammation of etiology other than GPA and healthy controls, and to identify those miRNAs that differentiate between clinically proven GPA patients with characteristic and non-specific GPA histological features in their sinonasal biopsy. Notably, we show that several aberrantly expressed miRNAs in biopsy tissue samples of sinonasal mucosa of patients with GPA are significantly associated with several histopathological and clinical parameters, and hold the potential for utilization as a supporting factor in establishing the diagnosis of GPA, which currently remains challenging especially in the early stage.

## 2 Materials and methods

### 2.1 Patients

This retrospective study included biopsy samples of sinonasal mucosa from 37 newly diagnosed patients with clinically proven active GPA, 15 patients with sinonasal inflammation of other etiology and 14 control patients with normal histology or very mild inflammation. Overall, 20/37 (54%) patients with GPA had characteristic GPA histological features in their sinonasal biopsy and were designated as group GPA(+/+), while 17/37 (46%) patients with GPA presented mild, moderate or severe chronic inflammation with or without acute exacerbation in their sinonasal biopsy and were designated as group GPA(+/-). In patients with inflammation of other etiology in their sinonasal mucosa (group nonGPA), the diagnosis of GPA was excluded after a thorough clinical and histopathological evaluation, and a six months follow-up. These patients were diagnosed with chronic rhinitis, chronic sinusitis or chronic rhinosinusitis with nasal polyps. The control group, designated as CTRL, included patients with normal histology of sinonasal mucosa, taken during corrective surgery for nasal septal deviations, or mild inflammation in the sinonasal tract biopsy, obtained mostly from marginal tissues of benign lesion biopsies (e.g., polyps).

Tissue samples were collected between December 1999 and November 2023. GPA diagnosis was based on clinical, laboratory, imaging and histological findings. In addition, all GPA patients fulfilled the American College of Rheumatology (ACR) 1990 criteria for the classification of Wegener’s granulomatosis (29) or the 2022 ACR/European Alliance of Associations for Rheumatology (EULAR) classification criteria for GPA (30). The study was approved by the National Medical Ethics Committee of the Republic of Slovenia on April 8^th^ 2020 [approval No. 0120-101/2020/5] and was performed in accordance with the Declaration of Helsinki.

### 2.2 Histopathology

Biopsy samples were fixed in 10% neutral buffered formalin, embedded in paraffin and stained with hematoxylin and eosin (HE). Additional methods were performed if needed, such as van Gieson-Weigert staining to assess the integrity of the elastic lamina in the vessel walls, and Ziehl-Neelsen, Auramine-Rhodamine, Grocott and Periodic acid Schiff (PAS) stains to exclude tuberculosis and mycotic infection. In cases suspicious for lymphoma, appropriate immunohistochemical analyses were performed. For the purpose of this study, biopsies were re-evaluated by three expert pathologists (MŽ, NZ and MV), with particular focus on histopathological features important for evaluation of sinonasal mucosa related to GPA: granulomas, vasculitis and necrosis. In typical cases, granulomas in GPA are usually loose and not closely packed as in sarcoidosis or tuberculosis. Necrosis has a patchy distribution, with serpiginous borders; it is usually basophilic, with a finely granular appearance. Vasculitis in GPA typically involves small- to medium-sized arteries and veins with any of the following features: fibrinoid necrosis, fragmentation of the elastic lamina, acute and chronic inflammation, and granulomas (31). Therefore, special attention was given to these histological characteristics when performing histopathological examinations.

### 2.3 RNA isolation

Total RNA was isolated from 4–12 10-µm thick sections of formalin-fixed, paraffin-embedded (FFPE) sinonasal tissue samples with an AllPrep^®^ DNA/RNA FFPE Kit (80234, Qiagen, Germany), according to the manufacturer’s protocol. Yield and purity of isolated RNA were assessed with a NanoDrop™ One spectrophotometer and with a Qubit™ RNA HS Assay Kit (Q32855) on a Qubit™ 3.0 Fluorometer (all Thermo Fisher Scientific, USA). Isolated RNA was stored at –80°C.

### 2.4 Reverse transcription and cDNA synthesis

Reverse transcription and cDNA synthesis was performed with a miRCURY LNA RT Kit (339340, Qiagen, Germany) in 10 µl reaction volumes, according to the manufacturer’s protocol. Each reaction mixture contained 2 µl of 5× miRCURY RT Reaction Buffer, 1 µl of 10× miRCURY RT Enzyme Mix, 0.5 µl of UniSp6 RNA spike-in, RNase-free water and 10 ng (1 ng/µl) of total RNA template.

### 2.5 miRNA expression profiling

Profiling of miRNA expression in sinonasal tissues was performed with a miRCURY LNA™ miRNA miRNome PCR Panel, Human Panel I+II, V5 (YAHS-312YE-8, Qiagen, Germany) and a miRCURY SYBR Green PCR Kit (339347, Qiagen, Germany) on the QuantStudio™ 7 Pro Real-Time PCR System (Thermo Fisher Scientific, USA), according to the manufacturer’s protocol. Expression profiling was performed on two cDNA pools generated for each patient group [GPA(+/+), GPA(+/-), nonGPA and CTRL], yielding a total number of eight cDNA pools. Overall, an individual cDNA pool was generated by mixing equal volumes of undiluted cDNA (1 ng/µl) from six sinonasal tissue samples. For the control patient group, undiluted cDNA (1 ng/µl) from five sinonasal tissue samples was used to obtain an individual CTRL cDNA pool. Profiling in each cDNA pool was performed separately, where 40 µl (40 ng) pooled cDNA was used for the analysis, according to the manufacturer’s protocol.

Raw data was analyzed using a DA2 Design and Analysis Software 2.6.0 (Thermo Fisher Scientific, USA), including amplification plot and melt curve plot analysis, with threshold set at 0.20 and with auto baseline. Data of miRNA assays with non-specific PCR products, poor technical replicates between cDNA pools and quantification cycle (Cq) values ≥ 37 were omitted from the analysis. UniSp3 assay Cq values were used for inter-plate calibration, according to the manufacturer’s protocol. Due to their determined stable and highly comparable expression between all eight cDNA pools, geometric means of Cq values of miRNA assays miR-320a-3p, miR-320b and miR-505-3p were used as reference endogenous controls for ΔCq calculation and data normalization (Cq_geomean_ range 27.98–28.63). The relative miRNA fold change was calculated with the 2^–ΔΔCt^ method (32).

### 2.6 Quantitative real-time PCR validation of miRNA expression

Quantitative real-time PCR (qPCR) analysis was conducted in accordance with the Minimum Information for Publication of Quantitative Real-Time PCR Experiments (MIQE) guidelines (33), on a QuantStudio™ 7 Pro Real-Time PCR System (Thermo Fisher Scientific, USA), with a miRCURY SYBR Green PCR Kit (339347, Qiagen, Germany) and miRCURY LNA miRNA PCR Assays (339306; Qiagen, Germany), according to the manufacturer’s protocol. Analyses were performed in 10 µl reaction mixtures. All qPCR reactions were performed in duplicate, and each qPCR reaction mixture contained 5 µl of 2× miRCURY SYBR Green Master Mix, 1 µl of PCR primer mix, 0.05 µl of ROX Reference Dye, 0.95 µl of RNase-free water and 3 µl (0.05 ng) of cDNA template. The UniSp6 primer assay was used as a reverse transcription positive control and as an inter-plate calibrator, and geometric means of Cq values of miRNA primer assays miR-320a-3p, miR-320b and miR-505-3p were used as reference endogenous controls for ΔCq calculation and data normalization. The relative miRNA fold change was calculated with the 2^–ΔΔCt^ method (32), employing primer efficiency-corrected Cq values, as previously described (34). Analysis was performed on sinonasal tissues of 20 patients from the GPA(+/+) group, 17 patients from the GPA(+/-) group, 15 patients from the nonGPA group and 14 controls from the CTRL group. miRNA primer assays included in the analysis are listed in **Supplementary Table S1**.

### 2.7 Statistical analysis

Statistical analyses were performed with IBM SPSS Statistics 27.0 software (IBM Corporation, USA) unless stated otherwise. Venn diagrams were generated using an online tool “Calculate and draw custom Venn diagrams” from Bioinformatics & Evolutionary Genomics (https://bioinformatics.psb.ugent.be/webtools/Venn/ [last accessed in November 2024]). In miRNA expression profiling experiments, the unpaired Student’s t-test was used to assess significance in miRNA expression levels (ΔCq values) between GPA(+/+), GPA(+/-), nonGPA and CTRL cDNA pools, and Pearson’s (*r*) correlation coefficients to evaluate correlations between the obtained ΔCq values. Heatmap with unsupervised hierarchical clustering was generated using the RStudio v1.1.456 software (RStudio, Inc., USA) with the heatmap.2 function from the gplots v3.2.0 package, employing negative ΔCq values for each miRNA per sample group (i.e., cDNA pool). The normality of data distribution in qPCR validation experiments was assessed using the Q–Q plots and the Kolmogorov-Smirnov and Shapiro-Wilk tests. Following a non-normal data distribution, a non-parametric Mann-Whitney *U* test was used to statistically assess differences in relative miRNA expression levels between patient groups. Obtained *p*-values were adjusted using the Bonferroni correction method, due to multiple comparisons performed (n = 6). Differences between patient clinical and histopathological parameters were evaluated using the Mann-Whitney *U* test, and association between miRNA expression levels and evaluated parameters in patients with GPA with Spearman’s (*ρ*) correlation coefficients. All tests were two-tailed, and a *p*-value of < 0.05 was considered statistically significant unless stated otherwise.

## 3 Results

### 3.1 Patient characteristics

Characteristics of patients enrolled in this study are presented in **Table 1**. The majority of patients from the GPA(+/+) group presented clinical signs typical for AAV with sinonasal involvement, including poor general condition, fever, elevated C-reactive protein (CRP) levels, weight loss and epistaxis. Serological testing confirmed the presence of ANCAs in all 20 patients, where 16/20 (80%) and 4/20 (20%) patients had PR3-ANCA-positive (c-ANCA) and MPO-ANCA-positive (p-ANCA) serology, respectively (**Table 1**). All patients from the GPA(+/-) group had also ANCA-positive serology, with 8/17 (47%) patients displaying PR3-ANCA-positive and 9/17 (53%) patients MPO-ANCA-positive status (**Table 1**). Overall, the number of patients with the PR3- and MPO-ANCA-positive status significantly differed between the GPA(+/+) and GPA(+/-) groups (*p* < 0.05) (**Table 1**). Clinical presentation in all 17 GPA(+/-) cases was that of a generalized systemic disease with suspected sinonasal involvement, based on clinical symptoms and signs (i.e., bloody and/or purulent nasal discharge, crusting of nasal mucosa, mucosal ulcers, nasal septum perforation and saddle nose deformity) or imaging findings (i.e., computed tomography (CT) or X-ray suggestive for sinusitis). Patients in the nonGPA group clinically presented chronic rhinosinusitis, nasal polyps and epistaxis, whereas subjects in the control CTRL group displayed very mild or no symptoms of the sinonasal tract, and had mostly breathing problems related to polyps or septal deviation.

**Table 1:** Patient characteristics.

Characteristic	GPA(+/+)	GPA(+/-)	nonGPA	CTRL
Number of patients	20	17	15	14
Gender (male/female)	11/9	9/8	11/4	11/3
Age at biopsy [years]	57 (26–86)	65 (28–88)	63 (13–74)	47 (25–60)
ANCA-positive serology; n (%)	20/20 (100)	17/17 (100)	NA	NA
PR3-ANCA-positive serology; n (%)	16/20 (80)*	8/17 (47)	NA	NA
MPO-ANCA-positive serology; n (%)	4/20 (20)*	9/17 (53)	NA	NA
Granulomas; n (%)	13/20 (65)***	0/17 (0)	NA	NA
Vasculitis; n (%)	8/20 (40)**	0/17 (0)	NA	NA
Necrosis; n (%)	18/20 (90)***	2/17 (12)	NA	NA

GPA, granulomatosis with polyangiitis; ANCA, anti-neutrophil cytoplasmic antibody; NA, not applicable; PR3, proteinase 3; MPO, myeloperoxidase.

Data are presented as median (range), unless otherwise specified. Data between GPA(+/+) and GPA(+/-) groups were evaluated using the Mann-Whitney *U* test. A *p*-value of < 0.05 was considered statistically significant (*p < 0.05; **p < 0.01; ***p < 0.001).

### 3.2 Histopathology

**Figure 1:**
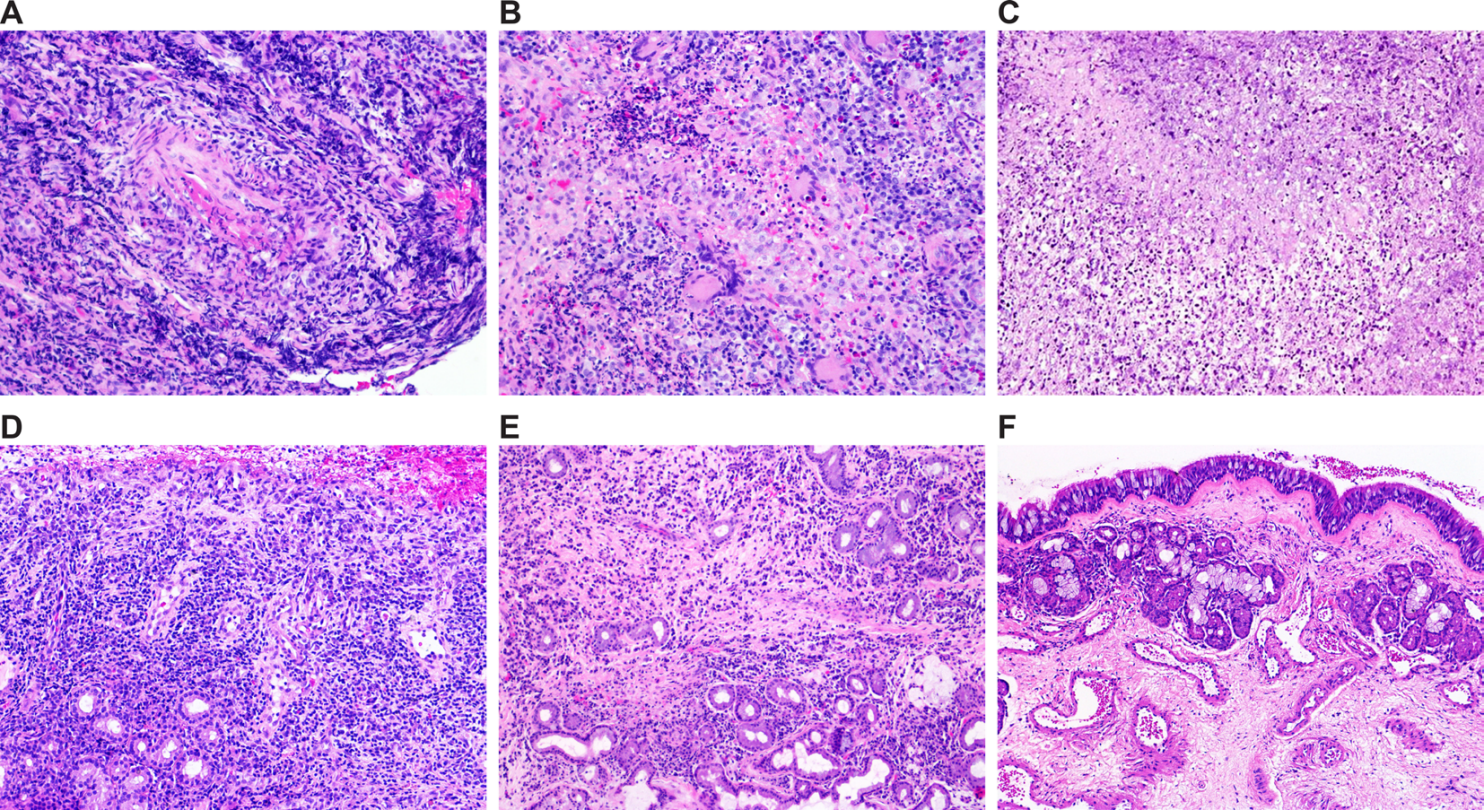
Selected representative images of histological features of sinonasal mucosa of patients included in the study. **(A–D)** Granulomatosis with polyangiitis: sinonasal mucosa with necrotizing vasculitis **(A)**, granulomatous inflammation and necrosis **(B)**, basophilic necrosis **(C)**, and non-specific inflammation **(D)**. **(E)** Sinonasal mucosa with inflammation of other etiology. **(F)** Healthy sinonasal mucosa. Original magnification: 20× **(A–E)** and 10× **(F)**.

Overall, patients in the group designated as GPA(+/+) showed different levels of small-vessel necrotizing vasculitis surrounded with mixed inflammatory infiltrate consisting of neutrophils, histiocytes, lymphocytes, plasma cells and, in some instances, multinucleated giant cells with foci of basophilic necrosis, characteristic for GPA. In contrast, patients in the GPA(+/-) group showed different morphological features, including mild, moderate or severe chronic inflammation or chronic inflammation with or without acute exacerbation and erosions. Accordingly, significant differences in the occurrence of histopathological features important for the evaluation of sinonasal mucosa related to GPA (i.e., granulomas, vasculitis and necrosis) were found between patients from the GPA(+/+) and GPA(+/-) groups (all *p* < 0.01) (**Table 1**). Histological features of patients from the nonGPA group were in some instances similar to those from the GPA(+/-) group, and were characterized by mild chronic inflammation with or without acute exacerbation. Subjects in the control CTRL group showed no histopathological changes in the tissue samples. Selected representative images of sinonasal mucosa of patients from the GPA(+/+), GPA(+/-), nonGPA and CTRL groups are presented in **Figure 1**.

As revealed by Spearman’s *ρ* correlation analysis, a significant positive correlation between the presence of necrosis and the presence of granulomas, vasculitis and PR3-ANCA-positivity was determined in 37 clinically proven patients with GPA (**Table 2**).

**Table 2:** Spearman’s correlation matrix of associations between evaluated parameters in patients with GPA.

	Gender	Age [years]^a^	PR3-ANCA^b^	Granulomas	Vasculitis	Necrosis
Gender	1					
Age [years]^a^	–0.203	1				
PR3-ANCA^b^	0.117	–0.208	1			
Granulomas	–0.117	–0.062	0.076	1		
Vasculitis	0.221	–0.237	0.090	0.017	1	
Necrosis	0.129	–0.175	0.585***	0.442**	0.319*	1

Spearman’s correlation coefficients (*ρ*) between evaluated parameters in patients with GPA (n = 37). A *p*-value of < 0.05 was considered statistically significant (*p < 0.05; **p < 0.01; ***p < 0.001) (marked in bold).

a Age at biopsy.

b Patients with PR3-ANCA-positive serology.

### 3.3 miRNA expression in patients and controls

**Figure 2:**
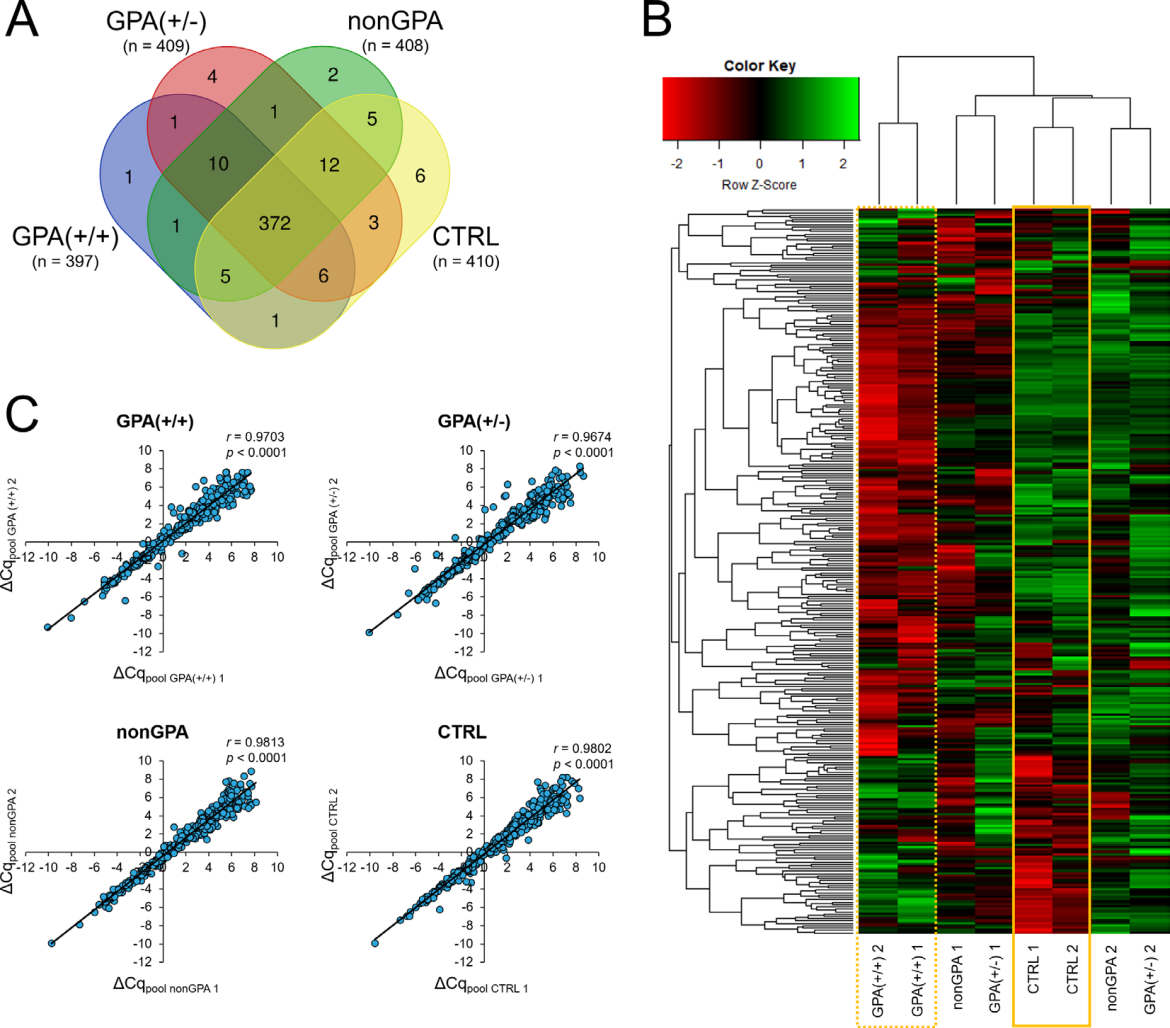
miRNA expression in GPA, non-GPA and control sinonasal tissues. **(A)** Venn diagram showing the number of identified miRNAs expressed in histologically positive clinically proven GPA patients [group GPA(+/+)], histologically negative clinically proven GPA patients [group GPA(+/-)], patients with inflammation of other etiology [group nonGPA] and patient controls [group CTRL]. **(B)** Heatmap with unsupervised hierarchical clustering of 306 miRNAs with determined expression in all eight cDNA pools and successful statistical evaluation (determined *p*-value) between the GPA(+/+), GPA(+/-), nonGPA and CTRL groups. Row Z-scores of negative ΔCq values are shown for each miRNA in every cDNA pool used in miRNA expression profiling experiments [GPA(+/+) 1, GPA(+/+) 2, GPA(+/-) 1, GPA(+/-) 2, nonGPA 1, nonGPA 2, CTRL 1 and CTRL 2]. The intensity of miRNA overexpression is shown in green and under-expression in red. The solid yellow square indicates miRNA expression levels in the control CTRL cDNA pools [CTRL 1 and CTRL 2], and the dotted yellow square miRNA expression levels in the GPA(+/+) cDNA pools [GPA(+/+) 1 and GPA(+/+) 2]. **(C)** Correlation (Pearson’s (*r*) correlation coefficients) between miRNA ΔCq values in each cDNA pool pair for every patient group [GPA(+/+), GPA(+/-), nonGPA and CTRL]. A *p*-value of < 0.05 was considered statistically significant.

**Figure 3:**
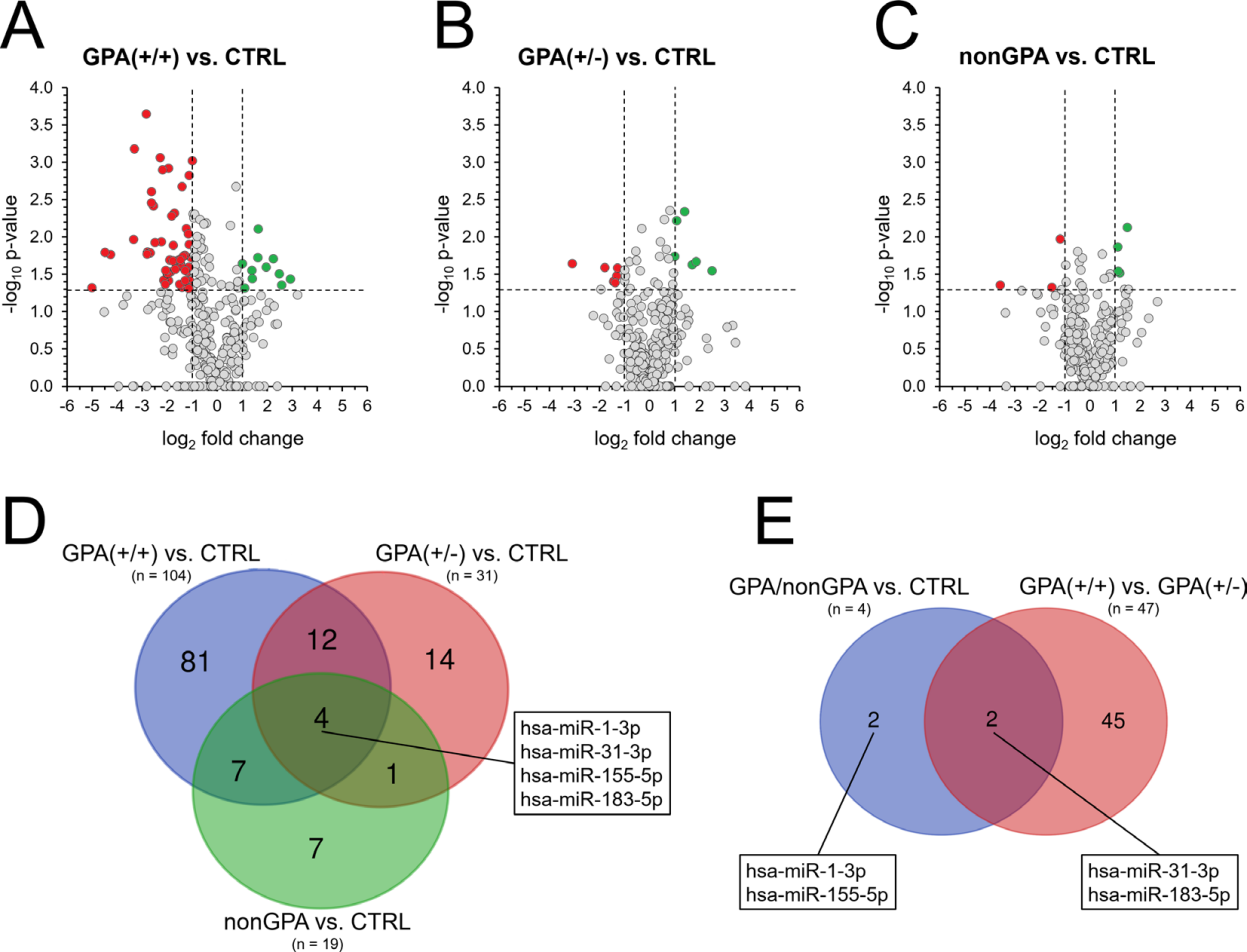
Alteration of miRNA expression in sinonasal tissues of GPA and non-GPA patients. Expression profiles of 369 miRNAs in patients from the GPA(+/+) group **(A)**, GPA(+/-) group **(B)** and nonGPA group **(C)**, all compared to the control CTRL group. Red dots represent miRNAs with significant > 2-fold under-expression and green dots miRNAs with significant > 2-fold overexpression. Horizontal line represents a *p*-value of 0.05 (unpaired Student’s t-test) and vertical lines the 2-fold miRNA fold change cut-off. Grey dots located directly on the x-axis represent miRNAs with undetermined *p*-value. **(D)** Venn diagram showing the number of miRNAs with determined significant differences in their expression profiles in groups GPA(+/+), GPA(+/-) and nonGPA, when compared to the control CTRL group. The box indicates four miRNAs that significantly discriminated sinonasal tissues of the control CTRL group from groups GPA(+/+), GPA(+/-) and nonGPA. **(E)** Venn diagram showing miRNAs from the box in figure panel **(D)** whose expression profiles also significantly differed between GPA(+/+) and GPA(+/-) patient groups. A *p*-value of < 0.05 was considered statistically significant.

**Figure 4:**
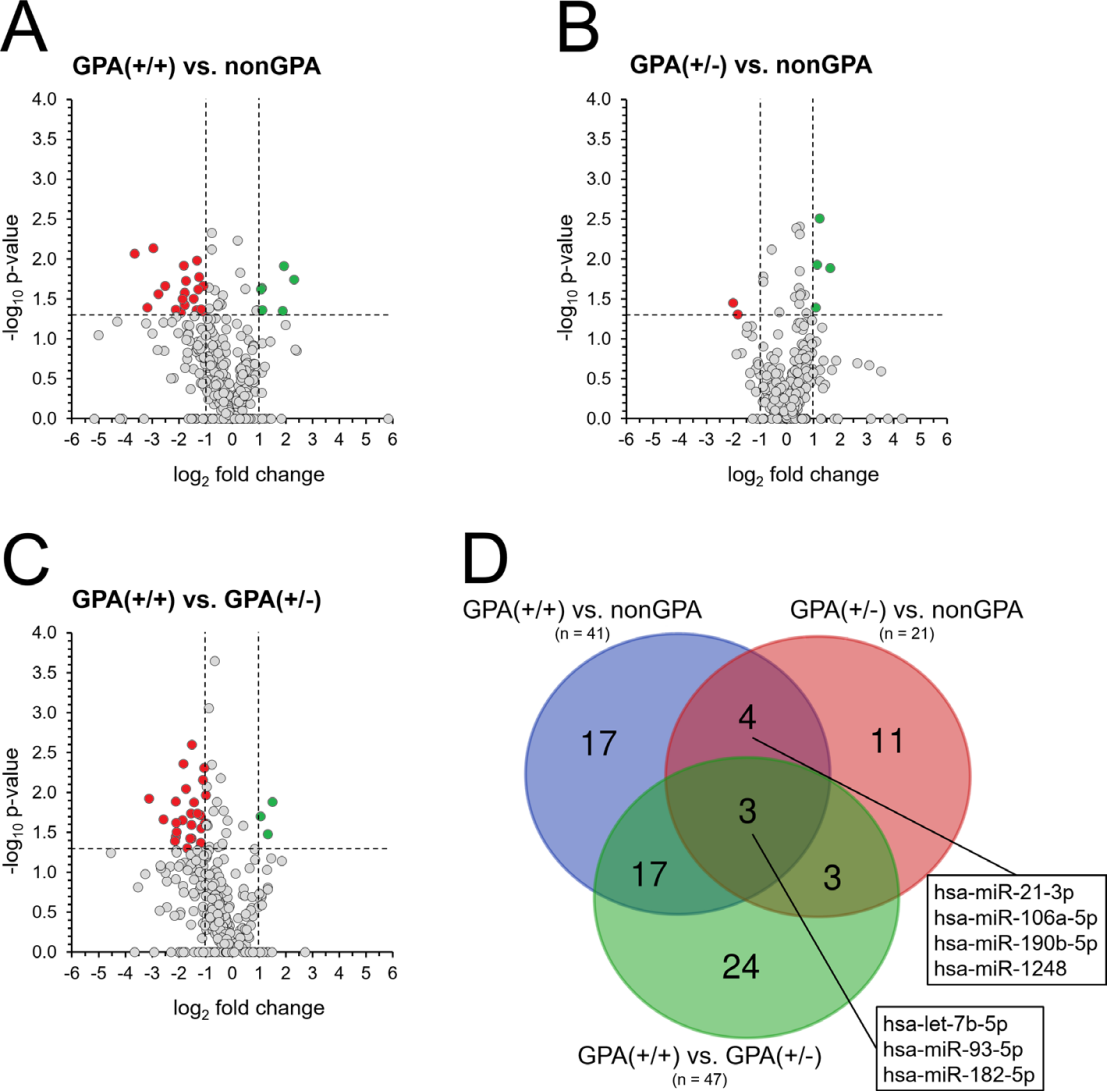
Differences in miRNA expression between non-GPA and GPA patient subgroups. Expression profiles of 369 miRNAs in patients from groups GPA(+/+) **(A)** and GPA(+/-) **(B)**, compared to the nonGPA group, and expression 369 miRNAs in patients from the GPA(+/+) group, when compared to the GPA(+/-) group **(C)**. Red dots represent miRNAs with significant > 2-fold under-expression and green dots miRNAs with significant > 2-fold overexpression. Horizontal line represents a *p*-value of 0.05 (unpaired Student’s t-test) and vertical lines the 2-fold miRNA fold change cut-off. Grey dots located directly on the x-axis represent miRNAs with undetermined *p*-value. **(D)** Venn diagram showing the number of miRNAs with significant differences in their expression profiles between sinonasal tissues of patients in the nonGPA, GPA(+/+) and GPA(+/-) groups. Boxes indicate four miRNAs that significantly discriminated between sinonasal tissues of GPA [GPA(+/+) and GPA(+/-)] and non-GPA patients, and three miRNAs whose expression profiles significantly discriminated between patients from the nonGPA, GPA(+/+) and GPA(+/-) groups. A *p*-value of < 0.05 was considered statistically significant.

Under our experimental conditions, expression of 430 miRNAs was detected in at least one of the eight cDNA pools [GPA(+/+) 1, GPA(+/+) 2, GPA(+/-) 1, GPA(+/-) 2, nonGPA 1, nonGPA 2, CTRL 1 and CTRL 2], from a total of 752 miRNA assays included in the miRCURY LNA miRNA miRNome Human PCR Panel I+II. Of these 430 miRNAs, we detected the expression of 397 miRNAs in GPA(+/+), 409 miRNAs in GPA(+/-), 408 miRNAs in nonGPA and 410 miRNAs in the CTRL cDNA pools (**Figure 2A**). As revealed, 372 miRNAs were expressed in all four patient groups (**Figure 2A**), in at least one cDNA pool. Since three miRNAs (miR-320a-3p, miR-320b and miR-505-3p) were used as endogenous controls for data normalization, expression profiles of an overall number of 369 miRNAs were determined (**Figures 3**, **4**; **Supplementary Tables S2–13**), of which, 306 miRNAs had determined expression in all eight cDNA pools (**Figure 2B**), and could be thus statistically evaluated between the control and patient groups. Excellent correlation scores in miRNA ΔCq values between each cDNA pool pair generated for every patient group (all *r* > 0.967; *p* < 0.001) (**Figure 2C**), confirmed the precision of miRNA expression profiling experiments.

### 3.4 miRNA expression is altered in sinonasal tissue of GPA and non-GPA patients

Differences in expression levels of 306 miRNAs between individual cDNA pools of the GPA(+/+), GPA(+/-), nonGPA and CTRL groups were initially assessed by unsupervised hierarchical clustering and displayed as a heatmap (**Figure 2B**). Notably, analysis revealed that both cDNA pools of the GPA(+/+) group clustered separately from cDNA pools of the GPA(+/-), nonGPA and CTRL groups (dotted yellow square in **Figure 2B**). Clustering also revealed that CTRL 1 and CTRL 2 cDNA pools formed a specific sub-cluster within a larger sub-cluster additionally comprising cDNA pools of the GPA(+/-) and nonGPA groups (solid yellow square in **Figure 2B**), indicating to a moderate degree of miRNA alteration in GPA(+/-) and nonGPA groups. Although perceivable differences in miRNA expression levels between cDNA pools of the GPA(+/-) and nonGPA groups were present, we determined an overall weaker degree of uniformity in miRNA expression between these four cDNA pools (**Figure 2B**).

When compared to the control CTRL group, 104 miRNAs were identified as significantly aberrantly expressed in sinonasal tissue of patients in the GPA(+/+) group (all *p* < 0.05), where 55 miRNAs were under-expressed > 2-fold and 11 miRNAs were overexpressed > 2-fold (**Figures 3A, D**; **Supplementary Tables S2, S3**). In contrast and in accordance with results of unsupervised hierarchical clustering (**Figure 2B**), miRNA alteration was less profound in sinonasal tissues of patients from the GPA(+/-) and nonGPA groups, in which 31 and 19 miRNAs were significantly aberrantly expressed (all *p* < 0.05), respectively (**Figures 3B–D**; **Supplementary Tables S4–S7**). As determined, six miRNAs were significantly under- and six overexpressed > 2-fold in the GPA(+/-) group, compared to the controls (**Figure 3B**; **Supplementary Tables S4, S5**), whereas three miRNAs were significantly under-expressed > 2-fold and four miRNAs were significantly overexpressed > 2-fold in the nonGPA group, compared to the control CTRL group (**Figure 3C**; **Supplementary Tables S6, S7**). Notably, comparison of miRNA expression between the GPA(+/+), GPA(+/-), nonGPA and CTRL groups revealed that expression profiles of four miRNAs, including miR-1-3p, miR-31-3p, miR-155-5p and miR-183-5p, enabled discrimination of the control CTRL group from the GPA(+/+), GPA(+/-) and nonGPA groups (**Figure 3D**). Of these four miRNAs, expression profiles of miR-31-3p and miR-183-5p also significantly differed between patients in the GPA(+/+) and GPA(+/-) groups (**Figure 3E**).

### 3.5 Altered miRNA expression profiles distinguish between non-GPA and GPA patient subgroups

Next, we aimed to identify miRNAs whose expression profiles discriminated between sinonasal tissue lesions of patients in the GPA and nonGPA groups. As determined, 41 miRNAs significantly differed between sinonasal tissues of patients from the GPA(+/+) and nonGPA groups (all *p* < 0.05), with 20 miRNAs under-expressed and six miRNAs overexpressed > 2-fold in the GPA(+/+) group, compared to the nonGPA group (**Figure 4A**; **Supplementary Tables S10, S11**). Furthermore, we identified 21 miRNAs whose expression profiles significantly differed between patients from the GPA(+/-) and nonGPA groups (all *p* < 0.05), with two miRNAs under-expressed and four miRNAs overexpressed > 2-fold in the GPA(+/-) group, when compared to the nonGPA group (**Figure 4B**; **Supplementary Tables S12, 13**). Overall, four miRNAs (miR-21-3p, miR-106a-5p, miR-190b-5p and miR-1248) significantly discriminated both GPA subgroups from the nonGPA group (**Figure 4D**).

Noteworthy, we revealed that expression of 47 miRNAs significantly differed between patients from the GPA(+/+) and GPA(+/-) subgroups (all *p* < 0.05). Of these miRNAs, 25 were under-expressed > 2-fold and three were overexpressed > 2-fold in the GPA(+/+) group, compared to the GPA(+/-) group (**Figure 4C**; **Supplementary Tables S8, S9**), indicating to crucial differences in the extent of miRNA alteration between these GPA patient subgroups, also apparent from a heatmap in **Figure 2B**. Moreover, when compared to miRNA expression status in the nonGPA patient group, three miRNAs (let-7b-5p, miR-93-5p and miR-182-5p) emerged, whose expression profiles significantly differed between GPA(+/+), GPA(+/-) and nonGPA patient groups (**Figure 4D**).

Based on the results of miRNA expression profiling experiments, 11 miRNAs (miR-1-3p, miR-31-3p, miR-155-5p, miR-183-5p, miR-21-3p, miR-106a-5p, miR-190b-5p, miR-1248, let-7b-5p, miR-93-5p and miR-182-5p) were selected for further qPCR validation, due to their potential to significantly discriminate between the controls, non-GPA and GPA patient subgroups (**Figures 3D**, **4D**).

### 3.6 Sinonasal miRNA signatures discriminate GPA and non-GPA patients from the control group

**Figure 5:**
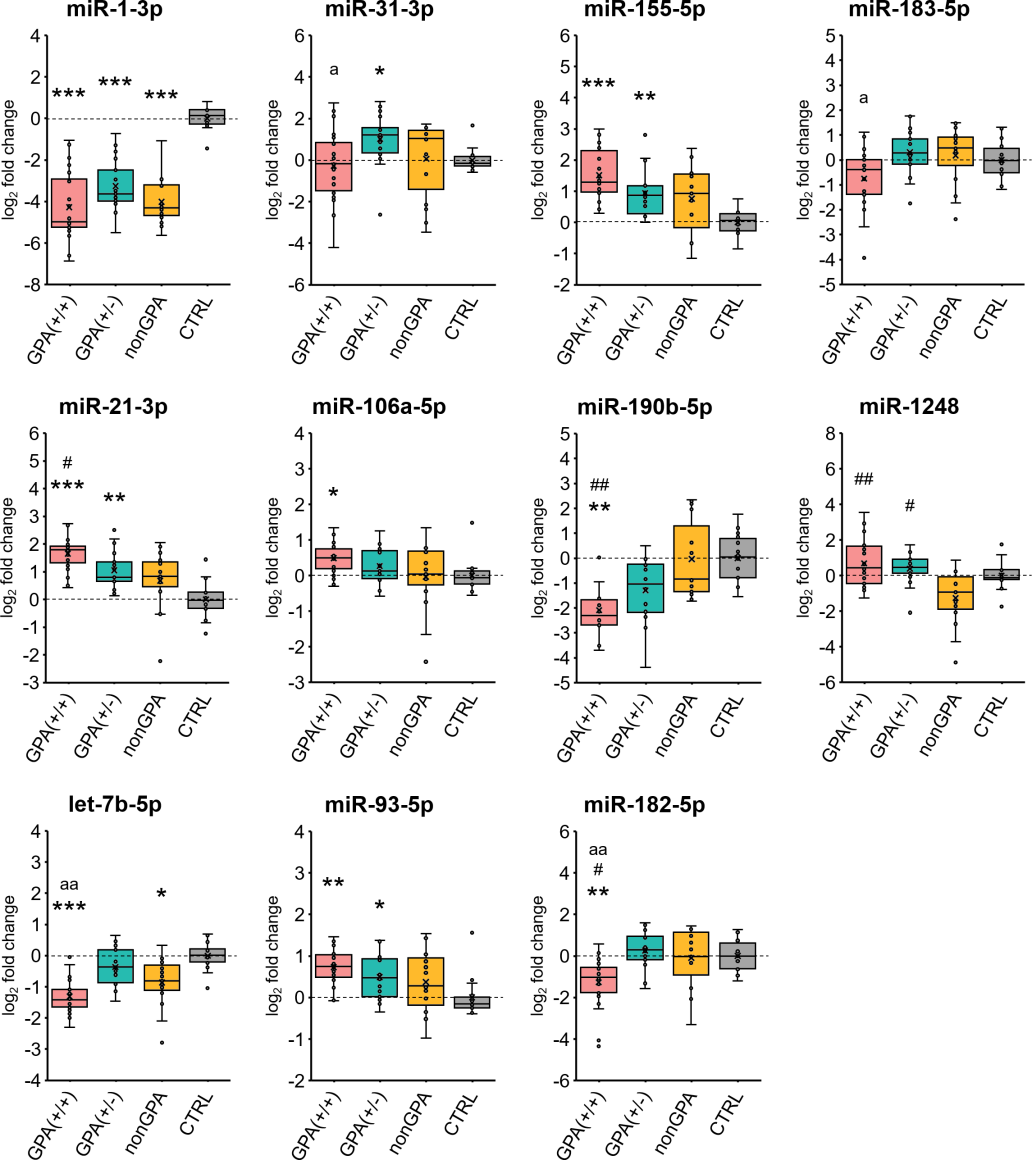
Validation of miRNA expression. Expression profiles of 11 selected miRNAs in sinonasal mucosa of patients from the GPA(+/+) [n = 20], GPA(+/-) [n = 17] and nonGPA [n = 15] groups, compared to the control CTRL patient group [n = 14]. The horizontal line within the boxplot denotes the median, and the horizontal borderlines denote the interquartile range. Each dot represents the log_2_ miRNA fold change of an individual sample. The symbol × denotes the average log_2_-fold change value. Data were evaluated using the Mann-Whitney *U* test. An asterisk indicates significance to the control CTRL group (*p < 0.05; **p < 0.01; ***p < 0.001), symbol # significance to the nonGPA group (^#^ *p* < 0.05; ^##^ *p* < 0.01), and the letter a significance to the GPA(+/-) group (^a^ *p* < 0.05; ^aa^ *p* < 0.01). An adjusted *p*-value of < 0.05 was considered statistically significant.

**Figure 6:**
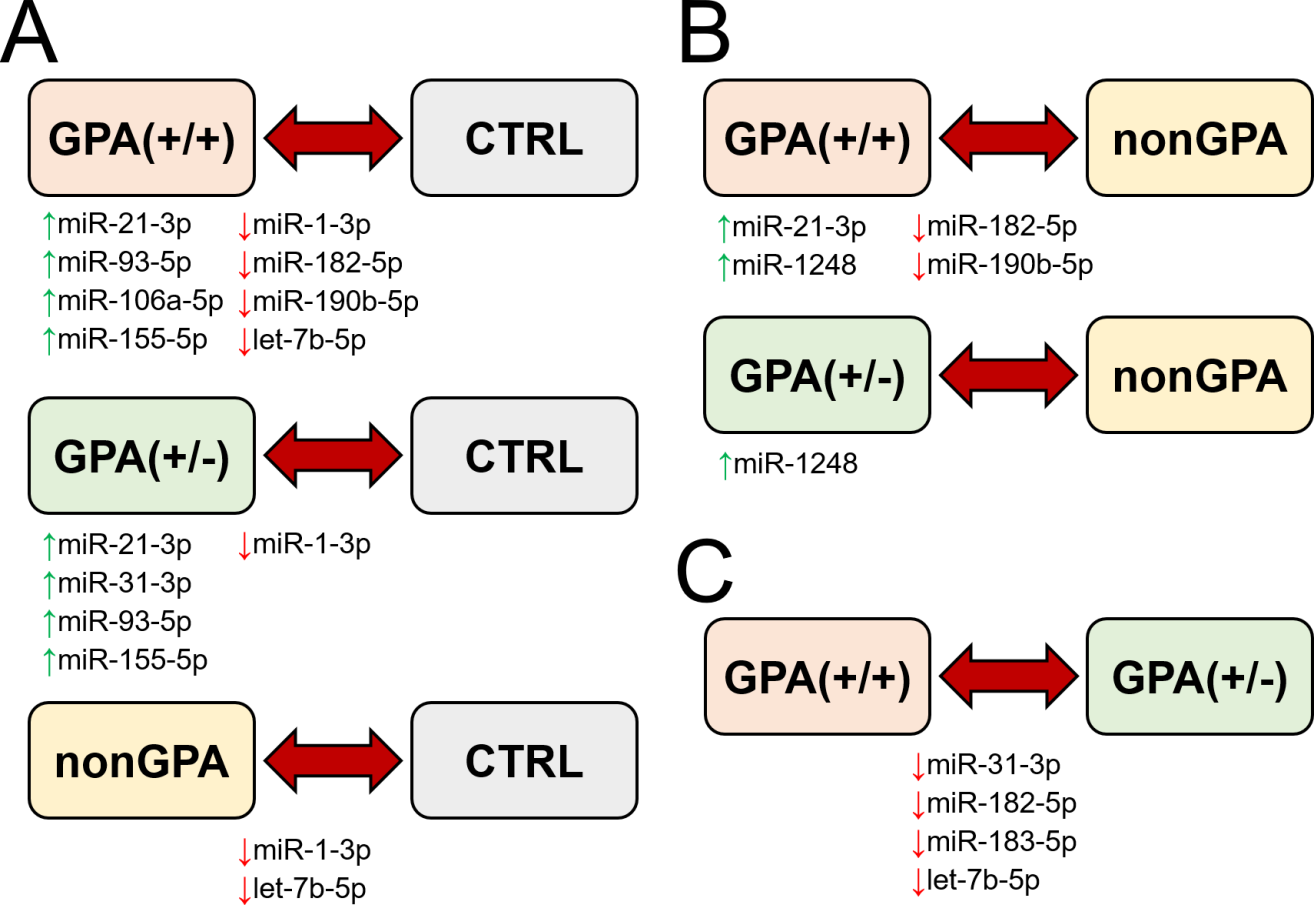
Schematic summary of miRNA expression validation in sinonasal mucosa of patients included in the study. Figure shows miRNAs with determined significant differential expression in patients from the GPA(+/+), GPA(+/-) and nonGPA groups, compared to the control CTRL group **(A)**, miRNAs with determined significant differential expression in patients from the GPA(+/+) and GPA(+/-) groups, compared to the nonGPA group **(B)**, and miRNAs with determined significant differential expression in patients from the GPA(+/+) group, compared to the GPA(+/-) group **(C)**. Green and red arrows indicate miRNAs determined as significantly overexpressed and under-expressed, respectively.

Validation qPCR experiments revealed that from the 11 selected miRNAs, expression profiles of eight miRNAs significantly differed between sinonasal mucosa of patients from the GPA(+/+) group and the control CTRL group (**Figures 5**, **6A**). Of these miRNAs, miR-21-3p/-93-5p/-106a-5p/-155-5p were overexpressed 1.4- to 3.2-fold (log_2_-fold change ranging 0.5–1.7; all *p* ≤ 0.012), and miR-1-3p/-182-5p/-190b-5p and let-7b-5p under-expressed 19.4- to 2.4-fold (log_2_-fold change ranging –4.3 to –1.3; all *p* ≤ 0.006), compared to the CTRL group. When expression of validated miRNAs was compared between patients from the GPA(+/-) and CTRL groups, we determined a significant 1.4- to 2.1-fold (log_2_-fold change ranging 0.5–1.1) overexpression of miR-21-3p/-31-3p/-93-5p/-155-5p and a significant 9.6-fold (log_2_-fold change –3.3) under-expression of miR-1-3p in the GPA(+/-) group, compared to the CTRL group (all *p* ≤ 0.024) (**Figures 5**, **6A**). As determined, miR-1-3p and let-7b-5p were the only significantly differentially expressed miRNAs in patients from the nonGPA group when compared to the CTRL group, and were under-expressed 16.1-fold (log_2_-fold change –4.0; *p* < 0.001) and 1.8-fold (log_2_-fold change –0.9; *p* = 0.012), respectively (**Figures 5**, **6A**).

Overall, expression profiles of four miRNAs (miR-1-3p/-21-3p/-93-5p/-155-5p) significantly discriminated patients with GPA [from both, GPA(+/+) and GPA(+/-) groups] from the control CTRL group, whereas miR-1-3p was the only miRNA to significantly discriminate sinonasal mucosa of the control cohort from patients in the GPA(+/+), GPA(+/-) and nonGPA groups (**Figures 5**, **6A**).

### 3.7 Sinonasal miRNA signatures discriminate between GPA and non-GPA patients

Next, we assessed differences in expression of validated miRNAs in sinonasal mucosa between patients with GPA and patients from the nonGPA group, and between patients from the GPA(+/+) and GPA(+/-) groups. Notably, we determined a significant differential expression of four miRNAs in the GPA(+/+) group, compared to the nonGPA group, of which miR-21-3p and miR-1248 were overexpressed 2.0-fold (log_2_-fold change 1.0; *p* = 0.018) and 3.9-fold (log_2_-fold change 2.0; *p* = 0.006), respectively. On the other hand, miR-182-5p and miR-190b-5p were under-expressed 2.3-fold (log_2_-fold change –1.2; *p* = 0.048) and 4.2-fold (log_2_-fold change –2.1; *p* = 0.002), respectively (**Figures 5**, **6B**). As revealed, miR-1248 was the only significantly differentially expressed miRNA in the GPA(+/-) group when compared to the nonGPA group, and was overexpressed 3.1-fold (log_2_-fold change 1.6; *p* = 0.012) (**Figures 5**, **6B**). Moreover, miR-1248 was also the only miRNA to significantly discriminate patients in both GPA(+/+) and GPA(+/-) groups from the nonGPA group (**Figures 5**, **6B**).

Noteworthy, we showed that expression profiles of miR-31-3p/-182-5p/-183-5p and let-7b-5p significantly differed between affected sinonasal mucosa of patients from the GPA(+/+) and GPA(+/-) groups, and were under-expressed 2.9- to 1.9-fold (log_2_-fold change ranging –1.5 to –0.9; all *p* ≤ 0.048) in the GPA(+/+) group, compared to the GPA(+/-) group (**Figures 5**, **6C**). Overall, miR-182-5p was the only validated miRNA whose expression profiles significantly discriminated patients in the GPA(+/+) group from CTRL, nonGPA and GPA(+/-) groups (**Figures 5**, **6**).

### 3.8 Interrelation between expression profiles of validated miRNAs and characteristics of patients with GPA

In order to interrelate expression profiles of 11 validated miRNAs with selected histopathological parameters in 37 patients with GPA, we statistically assessed the obtained data with Spearman’s *ρ* correlation analysis. Overall, we found a significant negative correlation between the expression levels of miR-1-3p and miR-31-3p and the presence of both vasculitis and necrosis (all *ρ* ≤ –0.32; *p* < 0.05), whereas expression of let-7b-5p significantly negatively correlated with all three evaluated histopathological parameters (granulomas, vasculitis and necrosis) (all *ρ* ≤ –0.43; *p* < 0.01) (**Table 3**). Moreover, expression levels of miR-21-3p/-155-5p/-182-5p significantly correlated with the presence of both granulomas and necrosis (all *p* < 0.05) (**Table 3**). As determined, expression of miR-93-5p and miR-106a-5p significantly positively correlated with the presence of granulomas (both *ρ* ≥ 0.33; *p* < 0.05), and expression of miR-183-5p significantly negatively correlated with the presence of necrosis (*ρ* = –0.35; *p* < 0.05) (**Table 3**). In addition, a significant positive correlation was found between the age and PR3-ANCA-positivity and expression levels of miR-190b-5p and miR-21-3p, respectively (**Table 3**).

**Table 3:** Spearman’s correlation matrix of associations between miRNA expression levels and evaluated parameters in patients with GPA.

miRNA	Gender	Age [years]^a^	PR3-ANCA^b^	Granulomas	Vasculitis	Necrosis
miR-1-3p	–0.097	0.297	–0.268	–0.233	–0.338*	–0.342*
miR-21-3p	–0.102	–0.059	0.411**	0.484**	0.104	0.439**
miR-31-3p	–0.320	0.271	–0.208	–0.142	–0.319*	–0.397*
miR-93-5p	0.046	0.116	0.215	0.330*	0.178	0.149
miR-106a-5p	0.041	0.031	0.169	0.416**	0.144	0.163
miR-155-5p	–0.229	–0.238	0.191	0.598***	–0.020	0.347*
miR-182-5p	–0.097	0.306	–0.169	–0.513**	–0.293	–0.492**
miR-183-5p	–0.065	0.273	–0.225	–0.314	–0.188	–0.352*
miR-190b-5p	–0.166	0.366*	–0.122	–0.248	–0.231	–0.268
miR-1248	–0.041	–0.203	–0.150	0.264	–0.059	–0.051
let-7b-5p	–0.264	0.288	–0.244	–0.515**	–0.426**	–0.563***

Spearman’s correlation coefficients (*ρ*) between expression levels of 11 validated miRNAs and evaluated parameters in patients with GPA (n = 37). A *p*-value of < 0.05 was considered statistically significant (*p < 0.05; **p < 0.01; ***p < 0.001) (marked in bold).

a Age at biopsy.

b Patients with PR3-ANCA-positive serology.

## 4 Discussion

The aim of our present study was to identify miRNAs whose expression profiles would help in the diagnostic assessment of patients in early stages of GPA or patients with atypical histological or clinical presentation, by revealing the distinctive miRNA expression patterns in sinonasal tissue samples in clinically and histologically proven GPA. Through the utilization of miRNA expression profiling, we initially uncovered 11 miRNAs (miR-1-3p/-21-3p/-31-3p/-93-5p/-106a-5p/-155-5p/-182-5p/-183-5p/-190b-5p/-1248 and let-7b-5p) with a potential to discriminate between GPA, nonGPA and control subgroups. Notably, validation experiments confirmed that of these miRNAs, expression profiles of eight miRNAs, five miRNAs and two miRNAs were capable to significantly distinguish the control group from patients in the GPA(+/+), GPA(+/-) and nonGPA groups, respectively, indicating their diagnostic potential. Furthermore, four miRNAs (miR-1-3p/-21-3p/-93-5p/-155-5p) demonstrated similar trends in their expression in both GPA patient subgroups [GPA(+/+) and GPA(+/-)], compared to the control group, suggesting these miRNAs might be implicated in the pathogenesis of GPA.

Studies have shown that miR-21-3p promotes differentiation, proliferation and activity of effector IL-17-producing T helper 17 (Th17) cells, T cell activation, and the development and apoptosis of Tregs in various autoimmune diseases (35). Moreover, miR-21 and miR-31 (the latter also significantly overexpressed in the GPA(+/-) group, compared to the CTRL group) concurrently influence Treg differentiation by altering the expression of the transcription factor Foxp3, essential for the proper development of this specific subset of CD4^+^ T cells (36). In addition to miR-21, miR-155-5p is regarded as a major pro-inflammatory miRNA, whose upregulation promotes classically activated M1-like macrophage responses, differentiation of effector CD4^+^ Th1 and Th17 cells, and production of cytokines that promote T cell-mediated autoimmune inflammation in a range of inflammatory conditions (19, 37). It has been demonstrated that miR-155 positively regulates CD8^+^ T cell responses (19, 38), which might also apply to miR-155 function in GPA, since it has been established that an increase in the number of effector CD8^+^ T cells plays an important role in tissue damage in AAV (39, 40). Overall, it is generally accepted that ANCAs generated by B cells represent the main contributors to the development of acute lesions in ANCA-associated diseases (5), whereas higher frequencies of peripheral blood Th17 cells and a lower percentage of Treg cells in an active GPA have been recognized as markers of disease activity in GPA (41). According to their determined expression and positive correlation with the presence of granulomas and necrosis in GPA-affected sinonasal mucosa, both miR-21 and miR-155 might be involved in the pathophysiology of GPA, implicated in Th17 cell-mediated innate inflammatory responses and granuloma formation, which have been highlighted over the years in acute phase of ANCA-associated diseases (42, 43). Nevertheless, further studies are needed to confirm these putative miR-21 and miR-155 functions in GPA.

In addition to miR-21 and miR-155, expression profiles of miR-93-5p and miR-1-3p also differentiated between patients with GPA and the control group in our study. As determined, miR-93-5p was significantly overexpressed and miR-1-3p under-expressed in both GPA patient groups, compared to the control CTRL group and their expression levels correlated with the presence of granulomas, and vasculitis and necrosis, respectively. Studies have shown that miR-93-5p regulates cell migration, invasion and proliferation through suppression of phosphatase and tensin homolog deleted on chromosome 10 (PTEN) in breast carcinoma and hepatocellular carcinoma (44, 45), whereas its function in GPA remains currently unexplored. Of note, epigenetic inhibition of PTEN has been shown to promote inflammation and activation of fibroblast-like synoviocytes in rheumatoid arthritis (46), and PTEN also functions as a modulator of neutrophil extracellular trap formation (NETosis) in circulating neutrophils, which is a prominent pathophysiological process in patients with GPA (47, 48). Nevertheless, further in-depth assessment is needed to interrelate the regulatory activity of miR-93-5p towards PTEN in GPA. Furthermore, an overexpressed miR-93-5p negatively regulates vascular endothelial growth factor A (VEGF-A) production in circulating peripheral blood mononuclear cells (PBMCs) in patients with Kawasaki disease, thus contributing to the pathogenesis of coronary artery involvement (49). However, since VEGF is abundant in sera of patients with GPA (50), such miR-93-5p function may not apply for GPA. Finally, dysregulation of miR-1-3p has been also linked to various disorders and production of pro-inflammatory cytokines (51, 52), although its role in GPA currently remains concealed.

Notably, our results show that a signature of four miRNAs (comprising miR-21-3p/-182-5p/-190b-5p/-1248) significantly discriminates sinonasal mucosa of patients with inflammation of etiology other than GPA from clinically proven GPA patients with characteristic GPA-related histopathological features. In contrast, miR-1248 was the only significantly differentially expressed miRNA in patients from the GPA(+/-) group, compared to patients from the nonGPA group, which was likely reflected by similar histological features of sinonasal biopsies between both patient groups, and was in accordance with the extent of miRNA alteration determined by our miRNA expression profiling experiments. Since miR-1248 was the only miRNA to significantly discriminate both GPA patient subgroups from the nonGPA group, our results indicate its putative biomarker potential in distinguishing GPA-related inflammation from other inflammatory conditions responsible for sinonasal lesion formation. Moreover, determined differential expression of miR-31-3p/-182-5p/-183-5p and let-7b-5p between sinonasal biopsies of patients from the GPA(+/+) and GPA(+/-) groups, suggests to crucial pathophysiological differences between GPA patients demonstrating different degree of the head and neck region involvement, presumably mediated by miRNAs involved in the pathogenic pro-inflammatory T cell responses and impaired Treg function (36, 53, 54).

Collectively, our results show that implementation of a multi-miRNA panel might hold a potential to stratify patients based on their sinonasal biopsy tissue miRNA profile, where: a) miR-1-3p/-21-3p/-93-5p/-155-5p may distinguish clinically proven GPA patients from healthy controls; b) miR-1248 may distinguish clinically proven GPA patients from patients exhibiting inflammation of other etiology; and c) miR-31-3p/-182-5p/-183-5p and let-7b-5p may distinguish between clinically proven GPA patients exhibiting characteristic and/or non-specific GPA-related histological features in their sinonasal biopsy. These nine miRNAs could thus serve as putative supporting biomarkers in establishing the diagnosis of GPA in patients in the early phases of the disease, or in patients with atypical presentation of GPA in their head and neck region.

To the best of our knowledge, only three studies investigating the role of miRNA dysregulation in GPA pathogenesis have included tissue samples of GPA-affected sinonasal mucosa, in combination with disease and/or healthy controls (26–28). However, when compared to our study, discrepancies in the number of identified dysregulated miRNAs have been observed. These variations could result from differences in the study design, the number and characteristics of enrolled patients, histopathology of the included biopsy material, patient treatment status and different pre-analytical procedures, which vary between laboratories and whose lack of standardization represents a major hurdle in implementation of miRNA-based biomarkers in clinical settings (55).

Overall, a limited number of enrolled patients represents the main constraint of our current single-center study. Expanding our analysis on a larger number of patients from various geographic regions would undoubtedly provide a more generalized insight into the role of miRNA alteration in GPA pathogenesis. Moreover, a comprehensive assessment of dysregulated miRNAs and their target gene networks in affected tissues and circulation of GPA patients with different organ involvement might reveal currently concealed miRNA-mediated mechanisms of GPA pathophysiology and facilitate the discovery of novel pathogenic determinants of GPA. In addition, utilization of digital PCR (dPCR) in determination of absolute copy numbers of individual miRNAs and/or a panel of miRNAs at limiting dilutions might hold a significant potential for accelerating miRNA-based biomarker discovery (55).

In summary, our study provides a novel insight into miRNA involvement in the pathogenesis of GPA, and shows that altered miRNA expression profiles in sinonasal mucosa differ between patients with clinically confirmed GPA, patients with inflammation of other etiology and healthy controls. Importantly, our results reveal that a panel of nine miRNAs comprising miR-1-3p/-21-3p/-93-5p/-155-5p/-1248/-31-3p/-182-5p/-183-5p and let-7b-5p holds the potential to stratify GPA and non-GPA patient subgroups based on their sinonasal tissue miRNA profile, and might serve as putative supporting biomarkers in establishing the diagnosis of GPA, particularly in patients with atypical presentation of GPA. Since our results suggest that many aberrantly expressed miRNAs in GPA associate with pathogenic T cell responses, further studies should address the complex interrelationship between miRNA dysregulation and tissue-damaging T cell-mediated autoimmune inflammation throughout the affected body areas in patients with AAV, which might eventually improve the management of AAV, including GPA.

## Data Availability

The original contributions presented in the study are included in the article/**Supplementary Material**. Further inquiries can be directed to the corresponding authors.
