# Supplementary material for: MicroRNA expression profiles in sinonasal biopsies to support diagnosis of granulomatosis with polyangiitis
Source: Front Immunol. 2025 Apr 14;16:1579750. doi: 10.3389/fimmu.2025.1579750 (PMC12034697; doi:10.3389/fimmu.2025.1579750)
Supplement: Supplementary file 1 [file Table1.docx]

**Supplementary Table S1.** miRNA primer assays.

| miRNA | Catalogue number^a^ |
| --- | --- |
| let-7b-5p | YP00204750 |
| miR-1-3p | YP00204344 |
| miR-21-3p | YP00204302 |
| miR-31-3p | YP00204079 |
| miR-93-5p | YP00204715 |
| miR-106a-5p | YP00204563 |
| miR-155-5p | YP02119311 |
| miR-182-5p | YP00206070 |
| miR-183-5p | YP00206030 |
| miR-190b-5p | YP00206031 |
| miR-1248 | YP00204253 |
| miR-320a-3p^b^ | YP00206042 |
| miR-320b^b^ | YP02119299 |
| miR-505-3p^b^ | YP00204214 |
| UniSp6^c^ | YP00203954 |

*^a^miRCURY LNA miRNA PCR Assay catalogue number (product number 339306, Qiagen, Germany).*

*^b^miRCURY LNA miRNA PCR Assay used as an endogenous control for data normalization.*

*^c^miRCURY LNA miRNA PCR Assay specific for amplification of UniSp6 RNA spike-in control, used as a reverse transcription positive control and as an inter-plate calibrator.*
